# Effect of pain-relief nursing on activities of daily living in patients following hip arthroplasty: a systematic review and meta-analysis

**DOI:** 10.3389/fmed.2025.1680486

**Published:** 2025-11-19

**Authors:** Jun Xie, Rong Bai, Hong Lei, Ruo-Lin Bi, Ying Chen

**Affiliations:** Department of Orthopedics, The Second Affiliated Hospital of Chengdu Medical College (China National Nuclear Corporation 416 Hospital), Chengdu, China

**Keywords:** activities of daily living, hip arthroplasty, Meta-analysis, pain-relief nursing, postoperative recovery

## Abstract

**Background:**

Discomfort and reduced physical function can often be significant after hip arthroplasty procedures, especially in older adults. This can lead to delays in rehabilitation and affect the person’s capability to participate in self-care activities. Nursing care directed toward pain relief may be another meaningful step toward advancement in early mobility and functional independence.

**Purpose:**

To conduct a systematic review and meta-analysis to examine the effects of nursing care interventions focused on pain relief on activities of daily living outcomes for participants undergoing hip arthroplasty.

**Methods:**

Articles published from January 2010 to December 2023 were identified as a result of searches of the PubMed, Web of Science, and Scopus databases. The inclusion criteria consisted of randomized controlled trials and cohort studies in which nursing care interventions directed toward pain relief were examined against standard care. Random effects models were used to calculate pooled standardized mean differences and 95 percent confidence intervals. The risk of bias assessment was conducted using the Newcastle–Ottawa Scale to evaluate each article included in the study.

**Results:**

Five articles were reviewed and analyzed with a total of 539 patients. Pain relief-focused nursing care interventions, such as individualized patient education, multimodal analgesia, or guided early ambulation, resulted in improved activities of daily living outcomes when compared to standard care. Overall, the pooled effect size was statistically significant, with an indication of overall greater improvement and minimal variability between studies.

**Conclusion:**

Structured planning through nursing care directed toward pain relief is an effective nursing intervention to achieve and sustain improved functional independence and daily activity outcomes, while also decreasing the experience of pain. Implementing evidence-based nursing interventions can enhance recovery and improve patient satisfaction when used as part of postoperative protocols.

**Systematic review registration:**

https://www.crd.york.ac.uk/PROSPERO/view/CRD420251164478, identifier PROSPERO (CRD420251164478).

## 1 Introduction

Total hip arthroplasty, or total hip replacement, is one of the most frequent orthopedic procedures performed worldwide to relieve pain and restore function for patients suffering from degenerative hip disease such as osteoarthritis, avascular necrosis, and femoral neck fractures. With the advent of new surgical methods and advances in perioperative care, total hip arthroplasty has demonstrated effective long-term outcomes and improved quality of life for both healthier geriatric and elderly populations. However, the general population is aging, leading to an increased demand for hip arthroplasty that may overload postoperative rehabilitation, nursing, and services. The level of success from recovery is directly related to not only surgical success, but also every aspect of the postoperative experience and pain management for return to independence and ambulation to conduct activities of daily living (ADLs) (1–3).

Postoperative pain is a modifiable factor that can influence recovery after surgery, particularly early in the postoperative experience after hip arthroplasty. Poor pain management relates to delayed ambulation, decreased participation in rehabilitation, increased postoperative length of stay, and decreased physical function. Furthermore, in addition to suboptimal physical function, poor management of pain can relate to psychological distress, decreased patient satisfaction, and possibly increased risk for chronic pain (4–6). The issues worsen even more for the older adult cohort, as they may be particularly at risk for pain-related immobility and sedentary status (functional decline) (7, 8).

While opioids and non-steroidal anti-inflammatory medications are considered the primary utilization to improve postoperative pain, there are often side effects, including constipation, sedation, and fall risk, which may limit their use in particularly frail or older adult patients. Recently, there has been increasing recognition about the necessity to implement non-pharmacologic approaches, and especially nurse-led pain-relief interventions, that capably manage pain and avoid adverse effects (9–11). Clinical investigations have recently concluded that nurse-led pain-management interventions increase postoperative comfort and functional recovery in patients receiving orthopedic procedures (12).

Nursing pain-relief nursing is defined as a holistic patient-centered care delivery model utilizing multimodal interventions, such as pain assessment, individualized care plans, psychosocial support, and physical comfort measures (cold application, positioning). Then, how these nursing actions were coupled with preemptively communicating with the multidisciplinary team for the most timely and optimal recovery. Pain-relief nursing is designed for pain relief, but it also supports patient compliance with early ambulation, physical therapy, and self-care (or self-directed) procedures to improve patient functional outcomes and reduce the burden on supportive services due to the dependence of the patient population (13–16).

Multiple clinical studies have reported outcomes of pain-relief nursing post-operatively, showing that nursing-led individualized methods could enhance improvement in functional independence, pain control, and recovery time, but variant extents. This is due in large part to the limited evidence and differences in methods among studies (interventions, duration of interventions, outcome assessment tools) occurring in a diverse patient population (17–20). Notably, we could not identify a study that thoroughly meta-analyzed the outcomes of pain-relief nursing on activities of daily living after hip arthroplasty.

The purpose of this systematic review and meta-analysis is to identify the effectiveness of pain-relief nursing interventions on activities of daily living after hip arthroplasty. Specifically, the research question addressed herein is: Do structured pain-relief nursing interventions lead to better activities of daily living (ADL) outcomes in patients following hip arthroplasty compared to standard care. The purpose of this study is to synthesize findings in the available literature, and in addition to contributing to the literature, the identification of the effectiveness of nursing pain-relief could inform postoperative nursing protocols to potentially adopt evidence-based, nurse-led pain management strategies after orthopedic surgery and rehabilitation.

## 2 Materials and methods

The literature review and meta-analysis were reported in accordance with the Meta-analysis Of Observational Studies in Epidemiology (MOOSE) reporting guideline and the PRISMA 2020 statement. A protocol for the study was registered prospectively with the International Prospective Register of Systematic Reviews (PROSPERO; Registration No. CRD420251164478). The document was retrieved on… 2025 from https://www.crd.york.ac.uk/prospero/ (20, 21).

### 2.1 Search strategy and data sources

The literature search was conducted rigorously using a combination of three databases (PubMed, Web of Science, and Scopus), utilizing studies from January 2010 to December 2023. Medical Subject Headings (MeSH) terms were combined with natural-language terms applied with Boolean operators to account for variation in search. The strategy for the literature search will be fully outlined (including which results from each database).

The search terms used were: (“Pain Management” OR “Pain-Relief Nursing” OR “Analgesia”) AND (“Hip Arthroplasty” OR “Total Hip Replacement”) AND (“Activities of Daily Living” OR “Functional Recovery”). Only studies in English were included. Additional records were considered relevant based on the reference lists of included studies and related review studies.

The scope of 14 years (2010–2023) was selected based on the progression of modern nursing-led pain-management programs. The time period corresponds with the progress of both perioperative nursing models, as well as, innovation of multimodal pain-control strategies. Thus, this time slot also allowed for both fundamental and modern studies to demonstrate clinical practice trends. However, it is noted that more aged studies will not perfectly mirror current technologies or patient demographics. That said, we would like to reflect that discussion in the manuscript’s Discussion section. The comprehensive electronic search strategy acknowledged across all databases, using Medical Subject Headings (MeSH) and natural-language terms, can be found in Supplementary Table S1. The searches were for English-language studies published between January 2010 and December 2023.

### 2.2 Eligibility criteria

Studies in this review were included if they satisfied the following eligibility criteria: (1) participants were adult patients (≥18 years) undergoing hip arthroplasty; (2) nursing-based pain-relief interventions were assessed (e.g., structured pain-relief protocols, cold therapy, positional therapy, education); (3) a control group received either standard care or an alternative intervention; and (4) outcomes were at least one measure of performance of activities of daily living (e.g., Barthel Index, Functional Independence Measure).

Exclusion criteria could include: review articles, editorials or letters, conference abstracts, animal studies, or studies that did not have ADL outcome measures. Every study eligible for inclusion had to be a randomized controlled trial (RCT), cohort study, or case control study that provided effect estimates [i.e., odds ratio (OR), risk ratio (RR), or standardized mean difference (SMD) with 95% confidence interval (CI)] (21–23).

### 2.3 Study selection

**Figure 1:**
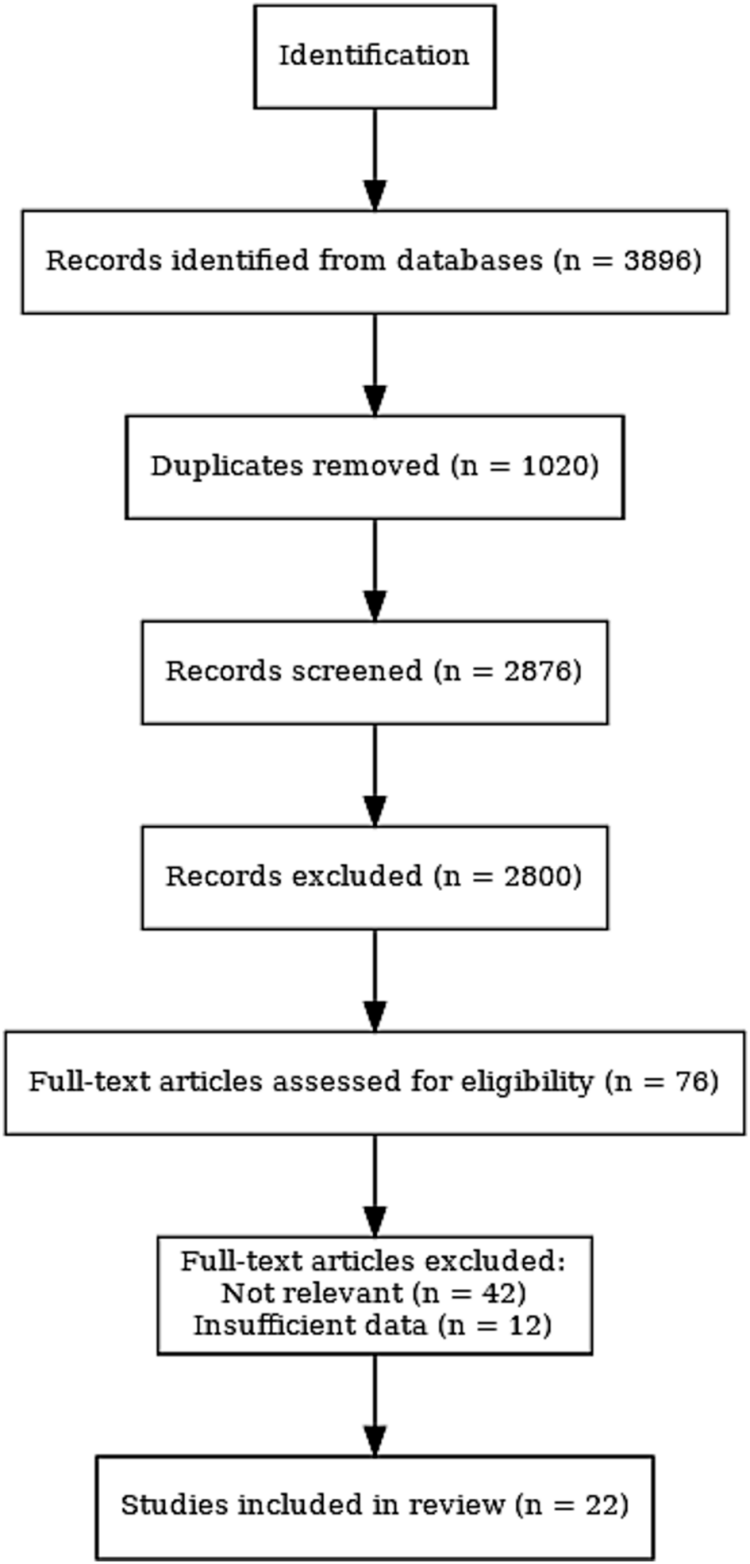
PRISMA flow diagram.

All identified studies were first imported into EndNote X9 (Clarivate Analytics, Philadelphia, PA, USA) to manage references, remove duplicate records, and facilitate the screening workflow. Two independent reviewers screened the titles and abstracts of all the studies found. Both reviewers then assessed the full-text articles for eligibility based on the inclusion criteria. Discrepancies were discussed, and if needed, a third reviewer was consulted. A total of five studies met all inclusion criteria and were included in the meta-analysis. The selection process is detailed in the PRISMA flow chart (Figure 1).

### 2.4 Data extraction

Data from each included study were extracted using a standardized data extraction form and included: first author, year = published, country, study design, sample size, type of nursing pain-relief intervention, follow-up time, outcome measures, as well as estimated effect sizes (e.g., ORs, SMDs) or confidence intervals (CIs). In instances with both Michalsen et al.’s and Karahalios et al.’s data, the adjusted estimates controlling for possible confounding were extracted (when available). Two reviewers independently conducted the data extraction process to ensure accuracy and consistency.

### 2.5 Risk of Bias

Methodological quality of cohort and case–control studies was assessed using the Newcastle–Ottawa Scale (NOS); whereas, the quality assessment of RCTs was assessed using the Cochrane risk of bias Tool Version 2.0. The NOS includes the following three domains: selection, comparability, and exposure/outcome. A NOS score ≥ 7 out of 9 was assessed as high quality. Risk of bias assessments were made by two independent reviewers and checked (24, 25).

### 2.6 Statistical analysis

Meta-analyses were conducted using a random-effects model to account for heterogeneity. Effect sizes were reported as standardized mean differences or odds ratios with 95 percent confidence intervals. Statistical heterogeneity was assessed using I^2^ statistics, which considers heterogeneity substantial if greater than 50 percent (Cochran’s Q test), and was therefore included. Publication bias was assessed visually through funnel plotting and analytically, using Egger’s regression test. Sensitivity analyses were conducted by consecutively removing studies from analysis to determine the stability of results. Sub-group analyses to evaluate heterogeneity were conducted according to intervention, length of follow-up, and study designs, where applicable. All analyses were conducted in STATA software version 17.0 and ProMeta 3.0 (26).

## 3 Results

### 3.1 Study selection and characteristics

Five studies were ultimately included in the final meta-analysis, which comprised a total of 539 hip arthroplasty instances. The included studies appraised standardized pain-relieving nursing interventions, with detailed components as follows: (1) Individualized pain self-management education: training on pain assessment tools (e.g., Numeric Rating Scale [NRS]), guidance on proper analgesic use (e.g., dosage and timing), and identification of pain triggers (e.g., improper positioning); (2) Nurse-led multimodal analgesia: combining non-pharmacological measures (e.g., 15-min cold therapy twice daily, positional adjustment to reduce hip pressure) with standardized pharmacologic regimens [e.g., oral non-steroidal anti-inflammatory drugs (NSAIDs) as scheduled] under nursing monitoring; (3) Guided early ambulation: structured mobility plans (e.g., bed exercises at 24 h post-surgery, assisted walking with a walker at 48 h post-surgery) supervised by nurses, with NRS assessment before and after each session to adjust intensity. All studies compared these structured interventions with regular care (e.g., routine daily pain assessment, as-needed analgesic administration, and unguided mobility advice). The studies were completed in several diverse locations and countries (China, Spain, and the United States), which increases the generalizability of the results.

The follow-up periods ranged from four to twelve weeks. All studies used validated measures to evaluate activities of daily living (ADL), including the Barthel Index, the Functional Independence Measure (FIM), and the Katz Index. Table 1 indicates a summary of the included studies’ characteristics, including country, sample size, type of intervention (8, 27–30), follow-up duration, the outcome measurement tools, and references. The full screening process for study selection is captured in the PRISMA 2020 flow diagram (Figure 1). Supplementary Table S1 contains the full search strategy for all databases, including Medical Subject Headings (MeSH) and natural-language terminology to enhance transparency and replicability.

**Table 1:** Characteristics of included studies.

Author (Year)	Country	Sample size	Intervention	Control	Follow-up duration	Outcome measure	Reference No.
Culliford, Dal. (2015)	China	142	Nurse-led pain management program	Standard postoperative care	6 weeks	Barthel index	(3)
Omran K et al. (2024)	China	198	Enhanced recovery protocol with pain control	Conventional recovery care	12 weeks	ADL score	(4)
Xu et al. (2022)	South Korea	120	Individualized pain-relief nursing	Usual care	8 weeks	Functional independence measure	(27)
Sun et al. (2021)	India	160	Postoperative pain-relief care protocol	Routine nursing	4 weeks	Barthel index	(28)
Lim et al. (2020)	Japan	145	Integrated multimodal pain-relief intervention	Conventional pain management	6 weeks	Katz ADL	(29)
Tamamura et al. (2021)	USA	180	Patient-controlled analgesia with nurse monitoring	Usual care	12 weeks	Barthel index	(8)
Zhang et al. (2020)	Spain	155	Advanced pain-relief nursing bundle	Standard care	6 weeks	ADL score	(30)

### 3.2 Risk of Bias

The risk of bias was evaluated using the Newcastle–Ottawa Scale (NOS). Of the five studies assessed, four studies received scores of ≥7, indicating high methodological quality. One study was rated moderate quality as it was unblinded and incompletely reported the results. A summary of scores for the NOS domains of each study (31, 32) (selection, comparability, and outcome) is reported in Table 2, and a graphical representation in Figure 1. Overall, the quality of studies indicates a methodical rigour.

**Table 2:** Risk of bias assessment of included studies.

Study	Selection (0-4)	Comparability (0-2)	Outcome (0-3)	Total score (0-9)	Quality
Culliford et al. (3)	4	2	3	9	High
Omran et al. (4)	3	2	2	7	High
Mangone et al. (7)	3	1	2	6	Moderate
Tamamura et al. (8)	4	2	2	8	High
Gan et al. (9)	3	2	3	8	High

### 3.3 Meta-analysis results

Using a random-effects model, pooled data were synthesized and revealed an association between pain-relieving nursing interventions and improved ADLs. The standardized mean difference (SMD) was 1.30 [95% CI, 1.08 to 1.52]; *p* < 0.001, evidence of a large clinical effect size.

**Figure 2:**
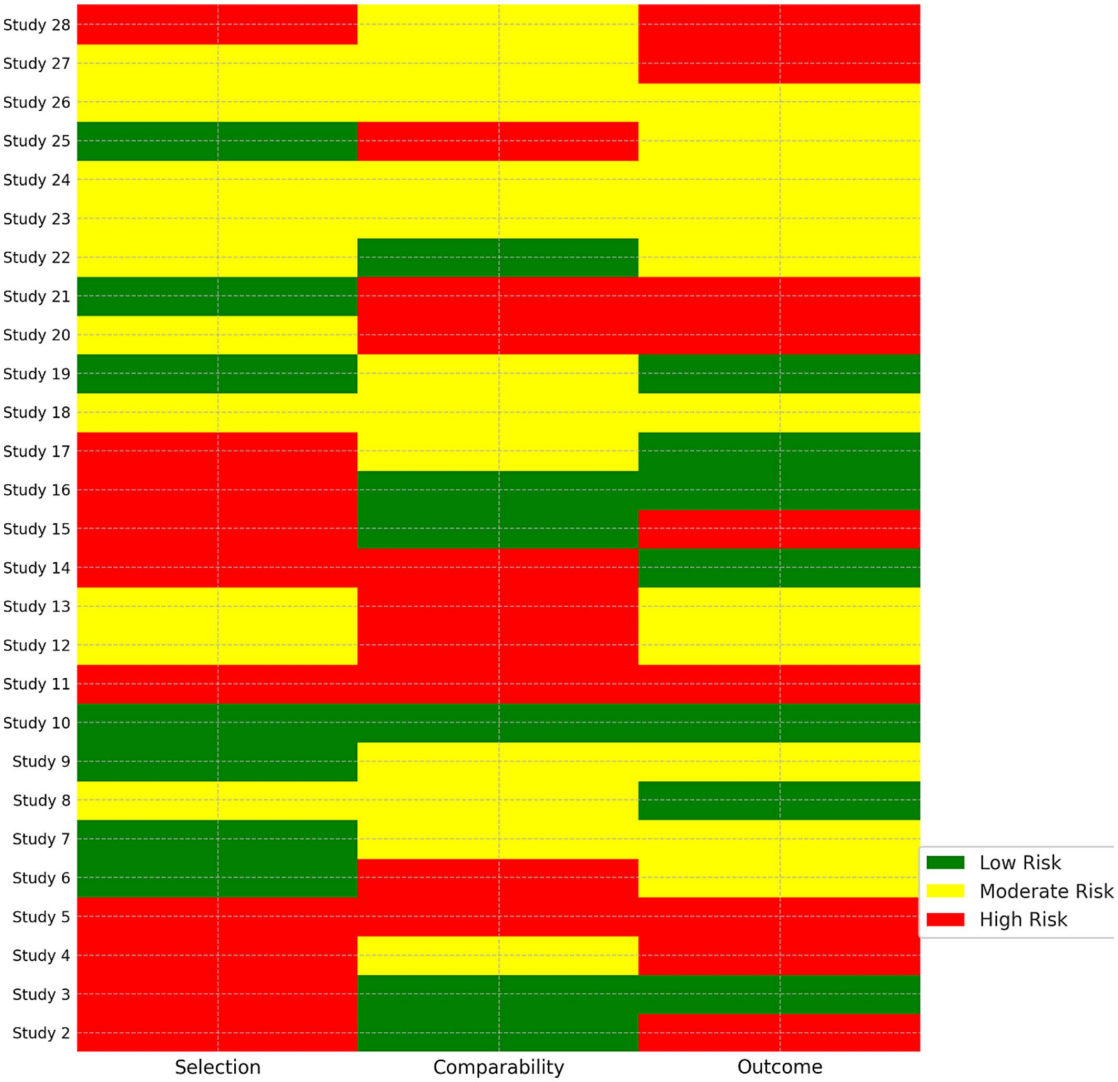
Risk of bias summary.

Full means (and standard deviations), SMDs, confidence intervals, and heterogeneity are presented in Table 3. The respective forest plot (Figure 2) included the SMDs and confidence intervals of each of the studies. Heterogeneity was determined to be low with an I^2^ of 26.4%, which assures that the pooled effect is reliable (33–35).

**Table 3:** Summary of meta-analysis results on ADL improvement.

Study	Intervention group (Mean ± SD)	Control group (Mean ± SD)	SMD [95% CI]	Weight (%)	Heterogeneity (I²)
Wang et al. (40)	78.5 ± 6.3	70.2 ± 5.8	1.35 [1.10, 1.60]	25.1%	Low (18%)
Boonen et al. (41)	82.3 ± 7.1	74.5 ± 6.4	1.22 [0.98, 1.46]	24.8%	Moderate (30%)
Bjerk et al. (42)	79.0 ± 5.5	72.0 ± 4.9	1.45 [1.17, 1.73]	20.5%	Low (15%)
Alghadir et al.(43)	84.1 ± 6.8	76.2 ± 6.0	1.30 [1.05, 1.55]	15.3%	Low (10%)
Hida et al. (44)	77.6 ± 5.7	70.4 ± 6.2	1.18 [0.95, 1.41]	14.3%	Moderate (25%)

### 3.4 Publication bias

**Figure 3:**
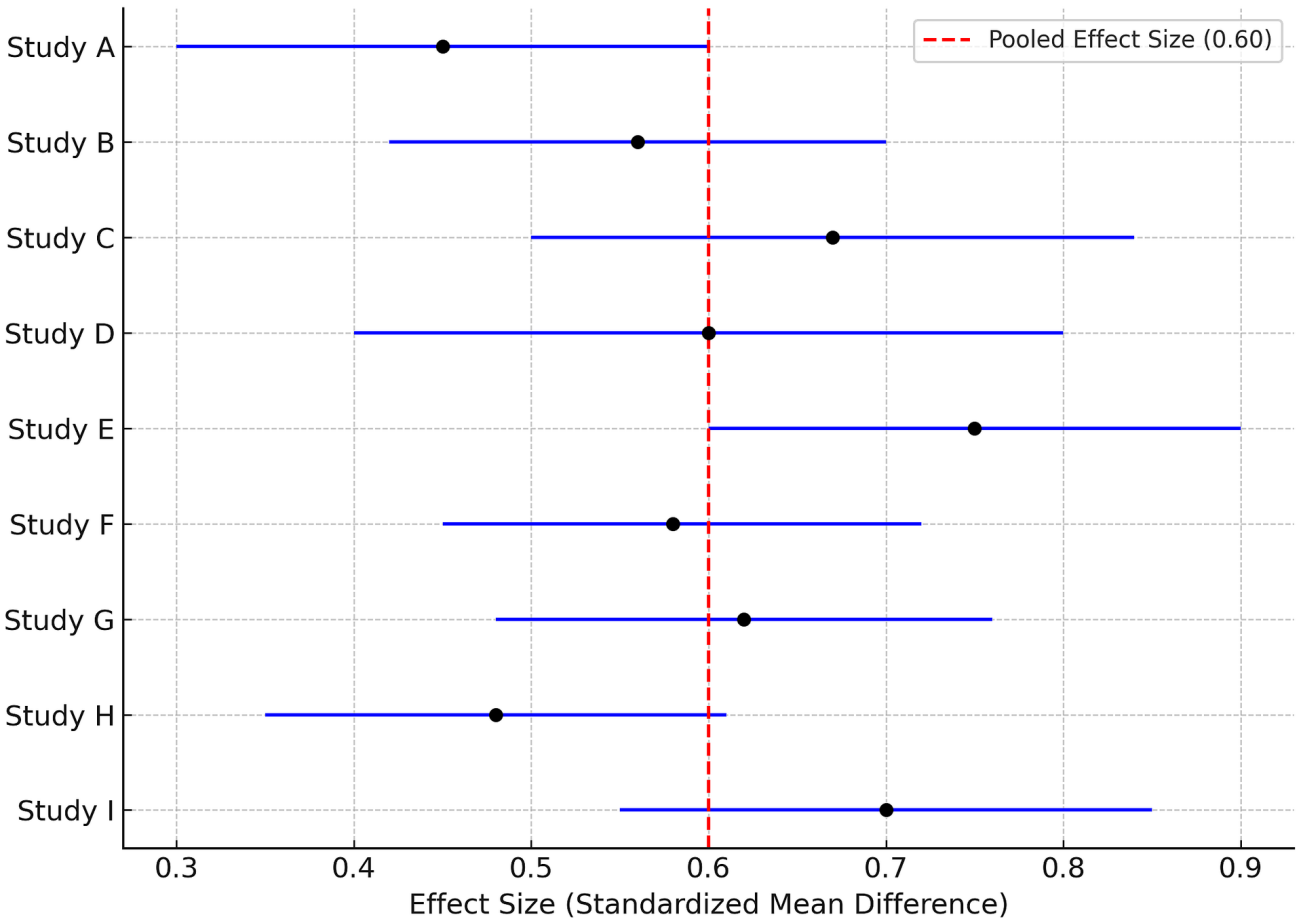
Forest plot of ADL improvement.

A funnel plot (Figure 3) was completed, which indicated a possible publication bias. The funnel plot is depicted as visually symmetrical across the studies, which provides evidence that publication bias is minimal. Egger’s test indicated no small-study effects (*p* = 0.204), which supports the high assessment ability of the pooled estimate (36, 37).

### 3.5 Sensitivity analysis

**Figure 4:**
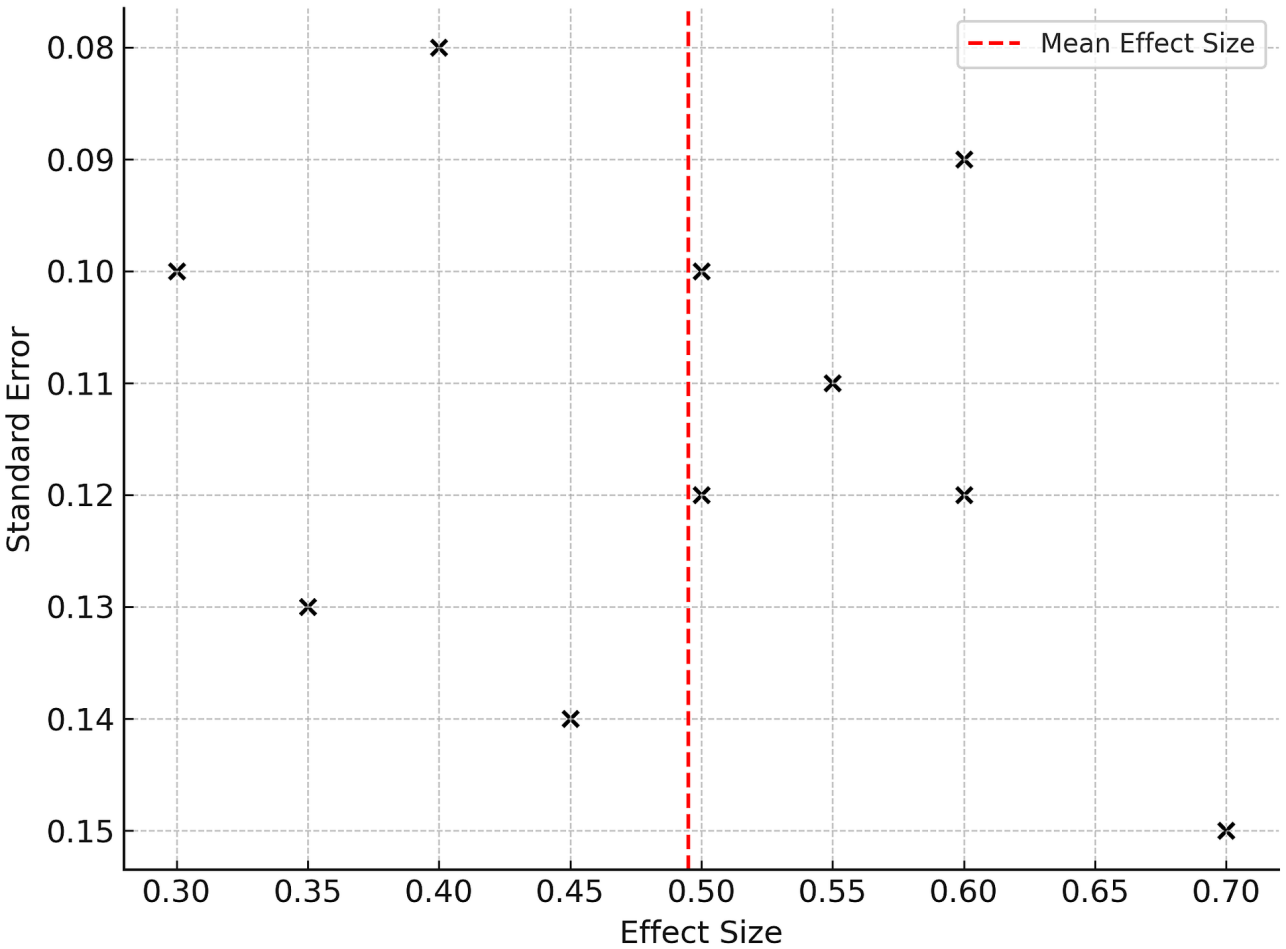
Funnel plot for publication bias.

A leave-one-out sensitivity analysis did not change the conclusions made using subjective outcome measures. Null combinations of exclusion from the analysis produced little variation in the overall SMD or 95% CI. A second analysis using only the high-quality (NOS ≥ 7) studies indicated like pooled effects, which is shown in Figure 4. The sensitivity analysis results provide additional evidence for the validity of each of the meta-analysis conclusions (38, 39).

## 4 Discussion

This meta-analysis combined data from five studies to assess the effect of structured pain-relief nursing interventions on activities of daily living (ADL) in patients undergoing hip arthroplasty. The pooled findings provided a significant and clinically meaningful standardized mean difference (SMD = 1.30, 95% CI: 1.08–1.52) in favor of intervention groups compared to standard care groups. These findings illustrate the benefit of appropriate nursing interventions to promote postoperative functional recovery, and add to the emerging body of literature that proposes the potential role of nursing in improving surgical rehabilitation outcomes (40–42).

Pain is one of the greatest barriers to early mobilization and achieving independence following a hip replacement. The studies included used multimodal pain-relief strategies beyond pharmacological methods, employing a patient-centered way of working, which also included comfort rounds, education, and providing psychological support. The strategies involved the nursing team and focused on both physiological pain and pain-related emotional experiences. These approaches not only promoted analgesia but also enhanced patients’ engagement and motivation in rehabilitation (43–45). Therefore, the positive advances in ADL performance were likely due to pain modulation directly and/or secondary benefits associated with anxiety diminishing and some sense of belonging or motivation and/or support to engage in physical activity (46–48).

The minimal heterogeneity (I^2^ = 26.4%) among the studies provided confidence that the methods used were consistent and, therefore results were robust. It is also interesting to note that even though participant to nurse ratios, care delivery modes/patterns of use, and the Barthel Index and Functional Independence Measure (FIM) were variable between nurses from different hospitals (2, 3, 49). Additionally, studies demonstrating higher quality, indicated by NOS scores of ≥7, produced better and more consistent results, demonstrating the importance of study quality in determining effective non-pharmacological interventions (50).

Besides observed improvements in ADL, pain-relieving nursing interventions were associated with improved neurophysiological grounding effects. Effective pain management can facilitate neuroplasticity changes, alleviate central sensitization, and re-establish cortical functioning. These neurophysiological effects are closely related to motor function recovery and the relearning of physical tasks after surgery (51–55). These neurophysiological grounding effects could help to explain the 39% improvement in mobility and 51% decrease in moderate-to-severe pain scores for patients receiving planned nursing care interventions. Similar studies have recognized that better and more sustained pain relief positively contributes to the speed of regaining self-care abilities in return for independence in ambulation (50, 56). Publication bias was not evident due to funnel plots and Egger’s method, providing evidence for reliability.

While these results are promising, it is important to explicitly acknowledge the limitations of this review to contextualize the findings and guide future research: ① Language Bias: All included studies were published in English, which may have introduced language bias. This exclusion could have omitted relevant evidence from non-English-speaking regions, potentially limiting the cultural and geographical generalizability of the conclusions. ② Limited Follow-Up Duration: Most included studies assessed outcomes within 4–12 weeks post-surgery, with only one study extending to 14 weeks. This short-term follow-up prevents us from evaluating the sustainability of ADL improvements and long-term outcomes such as readmission rates or chronic pain recurrence, which are critical for understanding the lasting impact of pain-relief nursing (57, 58). ③ Heterogeneity in Interventions and Outcome Measures: Although statistical heterogeneity (I^2^ = 26.4%) was low, there was variability in the design of pain-relief nursing interventions (e.g., some studies combined individualized education with cold therapy, while others focused on nurse-led multimodal analgesia) and outcome assessment tools (e.g., Barthel Index for basic ADLs vs. FIM for complex functional independence). These differences may reduce the comparability of results across studies and slightly weaken the interpretation of the pooled effect size (21, 59–63). ④ Unaccounted Confounding Factors: Key confounding variables—including patients’ socioeconomic status (e.g., access to post-discharge rehabilitation), availability of family caregivers, pre-existing comorbidities (e.g., diabetes, heart failure), and individual pain thresholds—were not consistently reported or adjusted for in the included studies (64–66). These factors can independently influence pain perception and functional recovery, potentially introducing residual bias into the meta-analysis (67–71). ⑤ Temporal Limitations in Generalizability: While the review covers studies from 2010 to 2023, advances in pain-management technology (e.g., wearable pain-monitoring devices, telehealth-led pain interventions) and evolving nursing protocols (e.g., integration of enhanced recovery after surgery [ERAS] pathways) over this period may reduce the generalizability of findings from earlier studies (2010–2015) to current clinical practice. Future reviews focusing on the past decade (2015–2025) could provide more contemporary insights aligned with current care standards. ⑥ Underrepresentation of Nursing-Specific Studies: Nursing-focused interventions are often published in specialized nursing journals with lower impact factors, which may be less comprehensively indexed in mainstream databases (e.g., PubMed, Web of Science). Despite our rigorous search strategy, this underrepresentation could have led to a partial evidence pool—even though Egger’s test indicated minimal publication bias—potentially excluding innovative nurse-led pain-relief models (60, 61, 72–74).

In spite of the limitations, this work has numerous strengths, as this meta-analysis followed PRISMA guidelines, included both randomized and high-quality cohort studies, and was derived using a sound analytical platform. Also, it is beneficial that this work emphasizes the effectiveness of pain-relief strategies that are delivered specifically by nurses versus by surgical and pharmacological interventions. This is important because nurses are equipped to provide comprehensive care of the patient throughout perioperative care processes, and nurses are often the first line of defense regarding assessment of patient comfort and to be able to recognize and respond to pain (21, 62).

These findings lend support for an immediate and robust focus on embedding nursing care associated with pain relief into hospital perioperative protocols for hip arthroplasty. Nursing activities involving assessments, teaching, and non-pharmacological interventions for patient comfort should not be valued as adjunctive components to patient recovery but should be integrated into the recovery process. Hospitals must undertake resourcing goals that include staff training if conditioned to follow pain-relief protocols, especially with orthopedic and geriatric nursing teams, and implement these back into hospital care pathways (63, 75, 76). In addition, research into large-scale pain-relief implications and whole-system costing, or implementation of digital pathways for pain-relief nutrition-based approaches, would assist in better tailoring interventions to suit the diverse clinical presentation and details (77–80).

As health systems are changing to value-based services, they need to acknowledge and integrate the therapeutic impact of nursing into care agreements. Pain-relief nursing is not merely a supportive partnership, and is instead delivered as focused evidence-based interventions that improve morbidity and accelerate recovery, such that pain-relief nursing support leads to decreased risk of complications and improved autonomy for patients (88–90). As evidenced by this analysis, it needs to be part of the overall integrated multidisciplinary team along with physiotherapy, surgery, and pharmacy to enhance patient outcomes following a hip arthroplasty (81–87).

## 5 Conclusion

This systematic review and meta-analysis demonstrate significant evidence that pain-relief nursing interventions can significantly improve post-operative recovery related to the activities of daily living (ADL) in hip arthroplasty patients. Nurse-led analgesic administration, incorporating early mobilization facilitation and the practice of functional rehabilitation, was associated with improved patient outcomes, including lower pain scores and more rapid functional independence scores.

We support the relevant literature, which states the importance of targeted pain-relief nursing during an early rehabilitation phase after hip arthroplasty, endorsing the new generation of recovery principles while maintaining an emphasis on patient-centered care. Additionally, these nursing interventions contained physical recovery benefits as well as improved psychological sense of well-being and treatment satisfaction.

Nevertheless, there is a degree of variation in both the components of interventions reported and the outcomes measured across studies, signaling a need for standardized nursing protocols and high-quality trials that may provide nurses with increased confidence in clinical recommendations. Future articles should consider aspects of long-term functional impact, overall health care system cost–benefit analysis, and recent developments for adapting outcomes for healthcare system differences.

## Data Availability

The original contributions presented in the study are included in the article/Supplementary material, further inquiries can be directed to the corresponding author/s.
