# Supplementary material for: Effect of pain-relief nursing on activities of daily living in patients following hip arthroplasty: a systematic review and meta-analysis
Source: Front Med (Lausanne). 2025 Nov 19;12:1680486. doi: 10.3389/fmed.2025.1680486 (PMC12673344; doi:10.3389/fmed.2025.1680486)
Supplement: Supplementary file 1 [file Table_1.docx]

# Supplementary Table S1. Full Search Strategy for All Databases

This table presents the complete electronic search strategy used for PubMed, Web of Science, and Scopus. The search was restricted to English-language publications from January 2010 to December 2023.

| Database | Search Date | Search Terms and Syntax | Results Retrieved |
| --- | --- | --- | --- |
| PubMed | July 12, 2025 | ("Pain Management"[MeSH] OR "Pain-Relief Nursing" OR "Analgesia") AND ("Hip Arthroplasty"[MeSH] OR "Total Hip Replacement") AND ("Activities of Daily Living"[MeSH] OR "Functional Recovery") | 523 |
| Web of Science | July 12, 2025 | (TS=("Pain Management" OR "Pain-Relief Nursing" OR "Analgesia")) AND (TS=("Hip Arthroplasty" OR "Total Hip Replacement")) AND (TS=("Activities of Daily Living" OR "Functional Recovery")) | 428 |
| Scopus | July 12, 2025 | TITLE-ABS-KEY("Pain Management" OR "Pain-Relief Nursing" OR "Analgesia") AND TITLE-ABS-KEY("Hip Arthroplasty" OR "Total Hip Replacement") AND TITLE-ABS-KEY("Activities of Daily Living" OR "Functional Recovery") | 296 |

Note: Search conducted on July 12, 2025, using both Medical Subject Headings (MeSH) and natural-language terms. Boolean operators were applied to combine concepts.
